# Neuroimmune and epigenetic involvement in adolescent binge ethanol‐induced loss of basal forebrain cholinergic neurons: Restoration with voluntary exercise

**DOI:** 10.1111/adb.12731

**Published:** 2019-02-18

**Authors:** Ryan P. Vetreno, John Peyton Bohnsack, Handojo Kusumo, Wen Liu, Subhash C. Pandey, Fulton T. Crews

**Affiliations:** ^1^ Bowles Center for Alcohol Studies, School of Medicine University of North Carolina at Chapel Hill Chapel Hill NC USA; ^2^ Center for Alcohol Research in Epigenetics, Department of Psychiatry University of Illinois at Chicago Chicago IL USA; ^3^ Jesse Brown VA Medical Center Chicago IL USA

**Keywords:** adolescence, alcohol, binge drinking, choline acetyltransferase, methylation, reversal learning

## Abstract

Binge drinking and alcohol abuse are common during adolescence and cause lasting pathology. Preclinical rodent studies using the adolescent intermittent ethanol (AIE; 5.0 g/kg, i.g., 2‐day on/2‐day off from postnatal day [P]25 to P55) model of human adolescent binge drinking report decreased basal forebrain cholinergic (ie, ChAT+) neurons that persist into adulthood (ie, P56‐P220). Recent studies link AIE‐induced neuroimmune activation to cholinergic pathology, but the underlying molecular mechanisms contributing to the persistent loss of basal forebrain ChAT+ neurons are unknown. We report here that the AIE‐induced loss of cholinergic neuron markers (ie, ChAT, TrkA, and p75^NTR^), cholinergic neuron shrinkage, and increased expression of the neuroimmune marker pNF‐κB p65 are restored by exercise exposure from P56 to P95 after AIE. Our data reveal that persistently reduced expression of cholinergic neuron markers following AIE is because of the loss of the cholinergic neuron phenotype most likely through an epigenetic mechanism involving DNA methylation and histone 3 lysine 9 dimethylation (H3K9me2). Adolescent intermittent ethanol caused a persistent increase in adult H3K9me2 and DNA methylation at promoter regions of *Chat* and H3K9me2 of *Trka*, which was restored by wheel running. Exercise also restored the AIE‐induced reversal learning deficits on the Morris water maze. Together, these data suggest that AIE‐induced adult neuroimmune signaling and cognitive deficits are linked to suppression of *Chat* and *Trka* gene expression through epigenetic mechanisms that can be restored by exercise. Exercise restoration of the persistent AIE‐induced phenotypic loss of cholinergic neurons via epigenetic modifications is novel mechanism of neuroplasticity.

## Graphical abstract

Adolescent intermittent ethanol (AIE), which models human adolescent binge drinking, causes a loss of basal forebrain cholinergic neurons through neuroimmune and epigenetic processes that persists into adulthood. Voluntary wheel running after AIE restored the AIE‐induced neuroimmune and epigenetic processes leading to recovery of basal forebrain cholinergic neurons. Exercise restoration of the persistent AIE‐induced phenotypic loss of cholinergic neurons via epigenetic modifications is novel mechanism of neuroplasticity.

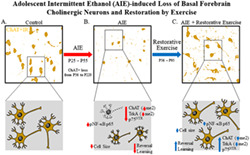

## 1 INTRODUCTION

Adolescence is an evolutionarily conserved developmental period of neurotransmitter system refinement that parallels the transition of the immature brain to the more efficient adult brain.^1^ The basal forebrain cholinergic system is essential for cognitive functioning^2^, ^3^ through its acetylcholine inputs to the cortex and hippocampus.^4^ Alcohol binge drinking is common during adolescence^5^, ^6^ and is associated with lasting consequences that persist into adulthood, including increased risk for diagnosis with alcohol use disorder,^7^ higher rates of comorbid mental disorders,^8^ and neuropathological changes in the basal forebrain.^9^ Studies using the preclinical rodent adolescent intermittent ethanol (AIE) model find diminished populations of choline acetyltransferase (ChAT)‐immunoreactive cholinergic neurons in the adolescent basal forebrain (ie, P56 [24 h post‐AIE]) which persist into adulthood (ie, P220 [165 days post‐AIE]^9^, ^10^). This is accompanied by somal shrinkage of the remaining ChAT+ neurons as well as persistent reductions of the high‐affinity nerve growth factor (NGF) receptor tropomyosin receptor kinase A (TrkA) and the low‐affinity NGF receptor p75^NTR^, which are highly expressed on basal forebrain cholinergic neurons^10^ and critical for cholinergic neuron function.^11^ The AIE‐induced loss of adult ChAT+IR neurons is associated with reversal learning deficits and persistent neuroimmune activation. Treatment with lipopolysaccharide induces forebrain neuroimmune genes and mimics the AIE‐induced loss of basal forebrain cholinergic neurons.^9^, ^10^ Exposure to voluntary wheel running or the anti‐inflammatory drug indomethacin during AIE treatment prevents ChAT+IR loss and neuroimmune gene induction.^10^ These findings are consistent with AIE neuroimmune gene induction contributing to adult ChAT+IR neuron degeneration.

Forebrain ChAT+ neurons are known to be dependent upon neurotrophic (eg, NGF) inputs from the cortex and hippocampus. Previous studies find fimbria‐fornix lesion‐induced loss of basal forebrain cholinergic neurons (ie, ChAT+ and p75^NTR^+) and cholinergic neuron shrinkage.^12^, ^13^ Recent studies suggest that AIE induces long‐lasting changes in neuroimmune genes as well as *Bdnf*, *Arc*, and other trophic‐neuroplasticity genes that are regulated through epigenetic mechanisms.^14^ For example, AIE exposure causes a long‐lasting inhibition of adult hippocampal neurogenesis that is reversed by exercise and anti‐inflammatory drugs^15^ as well as the histone deacetylase inhibitor TSA.^16^ Fimbria‐fornix lesion‐induced loss of basal forebrain cholinergic neurons (ie, ChAT+ and p75^NTR^+) and cholinergic neuron shrinkage can be recovered by intraventricular infusions of NGF.^12^, ^13^ Similarly, we previously reported that exercise exposure during AIE prevented the AIE‐induced loss of cholinergic neuron markers (ie, ChAT+, TrkA+, and p75^NTR^+) and cholinergic neuron shrinkage in the adult basal forebrain.^10^ These studies suggest that the persistent AIE‐induced loss of ChAT+ neurons may be because of the loss of the cholinergic phenotype, but the mechanism remains to be identified. Further, it is unknown if exercise exposure initiated in adulthood after adolescent binge ethanol exposure restores the AIE‐induced loss of cholinergic neurons and whether there is an epigenetic component that contributes to the loss of cholinergic neurons.

Emerging studies reveal that ethanol elicits chromatin remodeling in brain through epigenetic modifications leading to changes in gene expression that appear to contribute to alcohol‐induced neuropathology.^14^, ^17^ Epigenetic modifications involve histone acetylation and histone or DNA methylation, which can cause activation or repression of gene transcription without changing the underlying DNA sequence resulting in a specific phenotype.^18^, ^19^, ^20^ Interestingly, AIE treatment has been shown to induce long‐lasting epigenetic modifications in the amygdala and hippocampus of adult rats, and these effects can be prevented by administration of histone deacetylase inhibitors.^16^, ^21^ Acetylation and methylation of histone 3 lysine 9 (H3K9) is known to activate and repress gene transcription.^22^ Recently, it was reported that AIE increased histone 3 acetyl 9 dimethylation (H3K9me2) of *Bdnf* gene in the amygdala during adulthood.^23^ Interestingly, aerobic exercise has been shown to modulate epigenetic processes.^19^, ^24^ However, it is unknown if epigenetic processes contribute to the persistent loss of basal forebrain cholinergic neurons. We therefore tested the hypothesis that exercise exposure post‐AIE treatment (ie, P56‐P95) would restore cholinergic neuropathology. We report here for the first time that voluntary exercise exposure initiated 24 hours following AIE restored cholinergic neuron marker expression and blocked phosphorylation of proinflammatory NF‐κB p65 in the adult basal forebrain. We did not observe formation of new basal forebrain neurons following restorative exercise exposure consistent with loss of the cholinergic phenotype. Further, we report that AIE increased H3K9me2 and DNA methylation on promoter regions of the *Chat* gene and H3K9me2 on the *Trka* gene in the adult basal forebrain, which was prevented by wheel running exercise. In addition, wheel running restored the AIE‐induced reversal learning deficits on the Morris water maze. Together, these data implicate a novel neurobiological process involving neuroimmune and epigenetic mechanisms resulting in the phenotypic loss of basal forebrain cholinergic neurons following AIE.

## 2 MATERIALS AND METHODS

### 2.1 AIE paradigm

**Figure 1:**
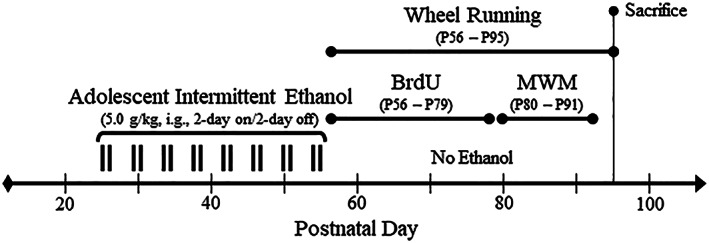
Graphical representation of the adolescent intermittent ethanol (AIE) paradigm and experimental design. On postnatal day (P)21, male Wistar rats were randomly assigned to either (1) water control (CON) or (2) AIE conditions. From P25 to P55, AIE subjects received a single daily intragastric (i.g.) administration of ethanol (5.0 g/kg, 20% ethanol, *w*/*v* [black tick marks represent a single ethanol binge]) in the AM on a 2‐day on/2‐day off schedule, and CON subjects received comparable volumes of water on an identical schedule. Tail blood was collected 1 hour after treatment to assess blood ethanol concentrations (BECs) on P38 (AIE/no exercise: 162 mg/dL [±13], AIE/exercise: 176 mg/dL [±54], one‐way ANOVA: *P* > 0.8) and P54 (AIE/no exercise: 137 mg/dL [±21], AIE/exercise: 159 mg/dL [±13], one‐way ANOVA: *P* > 0.4) using a GM7 analyzer (Analox, London, UK). Both CON‐ and AIE‐treated subjects were pair‐housed on P56 (24 h post‐AIE) in their respective housing conditions 24 hours per day in either standard cages (no exercise) or cages containing running wheels (exercise). Subjects ran a cumulative average of 242 km for the duration of experimentation (CON: 236 km [±14 km]; AIE: 249 km [±47 km]; one‐way ANOVA: *P* > 0.8). All subjects evidenced dramatic body weight increases across age (main effect of age: *P* < 0.01). We did not observe an effect of treatment (*P* > 0.9) or exercise (*P* > 0.05) on body weight although exercising subjects overall evidenced an approximately 10% reduction of body weight relative to the nonexercising subjects. 5′‐Bromo‐2′‐deoxyuridine (BrdU) was administered during restorative exercise exposure (75 mg/kg, i.p., every 4 days from P56 to P79) to determine generation of new cholinergic neurons in the adult basal forebrain. Spatial and reversal learning was assessed on the Morris water maze (MWM) from P80 to P91. At the conclusion of the study (P95), subjects were sacrificed and tissue collected for analysis

Male Wistar rats were used in this study. See Figure 1 for description of AIE treatment paradigm. Subjects were housed in a temperature‐ (20°C) and humidity‐controlled vivarium on a 12‐hour/12‐hour light/dark cycle (light onset at 0700 h) and provided ad libitum access to food and water. Experimental procedures were approved by the IACUC of the University of North Carolina at Chapel Hill and conducted in accordance with NIH regulations for the care and use of animals in research.

### 2.2 Voluntary wheel running

Both CON‐ and AIE‐treated subjects (n = 16‐20 subjects per group for immunohistochemistry [n = 8 subjects per group] and chromatin immunoprecipitation (ChIP)/methylated DNA immunoprecipitation (meDIP) [n = 8‐10 subjects per group] experiments) were pair‐housed on P56 (24 h post‐AIE) in their respective housing conditions 24 hours per day in either standard cages (no exercise) or cages containing running wheels (exercise). All groups were housed in pairs as social isolation can counteract the beneficial effects of exercise.^25^, ^26^ The exercise apparatus consisted of specially designed cages with a running wheel attachment (Panlab, Barcelona, Spain) connected to a LE3806 Multicounter (Panlab) allowing for collection of wheel revolutions.

### 2.3 5′‐Bromo‐2′‐deoxyuridine administration

To determine cell proliferation in the basal forebrain, subjects were treated with 5′‐bromo‐2′‐deoxyuridine (BrdU) (75 mg/kg, i.p. in sterile 0.9% saline; Sigma‐Aldrich, St. Louis, MO) every 4 days from P56 to P79.

### 2.4 Morris water maze

Spatial and reversal learning was assessed (n = 8‐10 subjects per group) as previously described.^27^ Briefly, habituation training was conducted one trial per day for two consecutive days beginning on P80. Twenty‐four hours later, spatial learning assessment was conducted over three trials per day for five consecutive days with an intertrial interval (ITI) of approximately 80 minutes per trail each day. The escape platform was situated in the middle of the northern quadrant 2 cm below the water line. Subjects were released into the water facing the wall at one of three pseudo‐randomized locations (ie, south, southwest, and southeast). Reversal learning assessment, which started 24 hours after the last spatial trial, was conducted over three trials per day for five consecutive days with an ITI of approximately 80 minutes. For the reversal learning trials, the escape platform was situated in the middle of the southern quadrant and placed 2 cm below the water line. Subjects were released into the water tank facing the wall at one of three pseudo‐randomized locations (ie, north, northwest, and northeast). A ceiling‐mounted automated tracking system (Ethovision XT 8.0, Noldus Ethovision; Leesburg, VA) was used to track the subject's movement.

### 2.5 Immunohistochemistry

At the conclusion of experimentation, subjects were anesthetized (n = 8 subjects per group), and tissue collected as previously described.^10^ Free‐floating basal forebrain samples (every sixth section; approximately Bregma: 1.60‐0.20 mm based on the atlas of Paxinos and Watson^28^) were processed as previously described.^10^ Briefly, sections were incubated in a primary antibody solution containing goat anti‐ChAT (Millipore, Temecula, CA, Cat. #AB144P), rabbit anti‐TrkA (Millipore, Cat. #06‐574), mouse anti‐p75^NTR^ (Millipore, Cat. #MAB365), mouse anti‐NeuN (Millipore, Cat. #MAB377), rabbit antiphosphorylated NF‐κB p65 Ser 536 (pNF‐κB p65; Abcam, Cambridge, MA, Cat. #ab86299), or mouse anti‐BrdU (Millipore, Cat. #MAB3424) for 24 hours at 4°C. For BrdU immunohistochemistry, DNA was additionally denatured by incubation in 2 N HCl for 30 minutes at 37°C followed by incubation in 0.1 M boric acid for 10 minutes at room temperature (pH: 8.5). The chromogen, nickel‐enhanced diaminobenzidine (Sigma‐Aldrich) was used to visualize immunoreactivity.

### 2.6 Microscopic quantification and image analysis

Across experiments, BioQuant Nova Advanced Image Analysis software (R&M Biometric, Nashville, TN) was used for image capture and quantification of immunohistochemistry as previously described.^10^ Briefly, a modified unbiased stereological quantification method was used to quantify immunopositive cells in the rat basal forebrain. The outlined regions of interest were determined, and data expressed as cells per square millimeter. Somal size was determined using BioQuant Nova Advanced Image Analysis software (R&M Biometric).

### 2.7 Fluorescent immunohistochemistry and microscopy

Free‐floating basal forebrain sections were processed as previously described.^29^, ^30^ Briefly, for assessment of cholinergic neuron marker colocalization, sections were incubated for 48 hours at 4°C in a primary antibody cocktail of goat anti‐ChAT (Millipore), TrkA (Millipore), and p75^NTR^ (Millipore). To assess cholinergic neuron marker colocalization with BrdU, tissue was similarly incubated in a primary antibody cocktail of goat anti‐ChAT (Millipore), rabbit anti‐NeuN (Millipore, Cat. #MABN140), and mouse anti‐BrdU (Millipore). Sections were then incubated for 2 hours at room temperature in the secondary antibody cocktail (rabbit Alexa Fluor 594, mouse Alexa Fluor 488, and goat Alexa Fluor 350; Invitrogen, Carlsbad, CA). Immunofluorescent images were obtained using a DS‐RiZ scope (Nikon Inc., Melville, NY) and colocalization quantified using NIS Elements AR46 (Nikon Inc.).

### 2.8 Chromatin immunoprecipitation

On P95, exercising and nonexercising CON‐ and AIE‐treated subjects (n = 8‐10 subjects per group) were anesthetized, and basal forebrain tissue dissected according to the atlas of Paxinos and Watson,^28^ rapidly frozen in liquid nitrogen, and stored at −80°C for ChIP assessment. The procedure is similar to the methods reported previously.^23^ Briefly, basal forebrain tissue samples were homogenized, fixed in 1.0% methanol‐free formaldehyde, quenched with 1.0 M glycine, lysed with lysis buffer (1.0% [*v*/*v*] SDS, 10 mM EDTA, 50 mM Tris‐HCl [pH 8.0]), and chromatin sheared to fragments of <1000 bp on a Covaris ME220. Input DNA fractions were removed from the sheared chromatin to be processed separately, and the remaining sheared chromatin was incubated overnight at 4°C with an antibody against H3K9/14Ac (Millipore, Cat. #06‐599), H3K9Ac (Active Motif, Carlsbad, CA, Cat. #39137, or H3K9me2 (Abcam, Cat. #ab1220). Protein A Dynabeads (ThermoFisher Scientific, Austin, TX) were added and rotated at 4°C for 1 hour followed by five washes in ChIP wash buffer. Both immunoprecipitated DNA and input DNA were eluted in 10% (*w*/*v*) Chelex by boiling at 95°C for 10 minutes followed by centrifugation. The resulting DNA was quantified using qPCR with SSOAdvanced Universal SYBR Green Supermix (Bio‐Rad, Berkeley, CA) using primers targeted against the *Trka* and *Chat* promoters (see Table 1). The ΔΔCt method was used to determine fold change relative to control and was normalized to the Input DNA fraction.

**Table 1:** List of primers for ChIP and meDIP analysis

Primer	Forward	Reverse	Target
*Chat* CpG promoter	TGCATCTGGAGCTCAAATCGT	GGGGATAGTGGTGACGTTGT	Promoter CpG island
*Chat* promoter	ACTTGATTGCTGCCTCTCTC	GGGATGGTGGAAGATACAGAAG	Promoter
*Chat* exon 2	GCTTAGGACACCCTTCATCTT	GCCCAGGATATTTACCAACACC	CpG island after exon 2
*Trka* promoter	CCTCACCGTGCACTTTACCT	AGGGTCTGGAGAGCGTACAT	Promoter (proximal)
*Trka* CpG promoter	TCAAGCAAGGCTCCGAACAG	CACAGGGTGGCGCTAGAAG	Promoter CpG island
*Trka* promoter	AGCATCGATTTCTGTGCGGA	CGTGACACGTATGCTTGCAG	Promoter (distal)

### 2.9 Methylated DNA immunoprecipitation

Frozen basal forebrain tissue (n = 8‐10 subjects per group) was processed using a DNeasy Blood & Tissue Kit (Qiagen, Hilden, Germany) to obtain DNA. The resulting DNA was fragmented to 200 to 500 bp, and DNA cleaned using a QIAquick PCR Purification Kit (Qiagen). DNA (1.0 μg) was then used for meDIP using the Methylated‐DNA IP Kit (Zymo, Irvine, CA, Cat. #D5101) following manufacturer's instructions. Following elution, meDIP and input DNA were quantified using qPCR with SSOAdvanced Universal. SYBR Green Supermix using primers targeted against the *Trka* and *Chat* promoters (see Table 1). The ΔΔCt method was used to determine fold change relative to control and was normalized to the input DNA fraction.

### 2.10 Statistical analysis

Statistical analysis was performed using SPSS (Chicago, IL). One‐way analysis of variance (ANOVA) was used to assess BECs and the NeuN immunohistochemistry data. The data on body weight and Morris water maze behavior were assessed using repeated measure ANOVAs. The immunohistochemical, ChIP, and meDIP data were analyzed using 2 × 2 ANOVAs. Post hoc analyses were performed using Tukey's HSD where appropriate. All values are reported as mean ± SEM, and significance was defined as *P* ≤ 0.05.

## 3 RESULTS

### 3.1 Voluntary wheel running restores AIE‐induced cholinergic neuropathology in the adult basal forebrain

**Figure 2:**
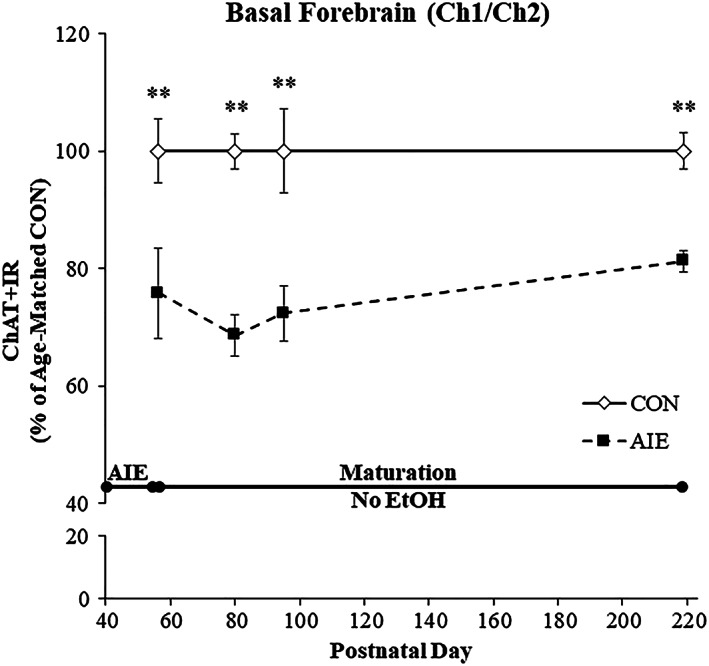
Adolescent intermittent ethanol (AIE) exposure reduces choline acetyltransferase immunoreactive (ChAT+IR) neuron populations in the late adolescent basal forebrain that persists into adulthood. Modified unbiased stereological assessment of ChAT+IR neurons in the basal forebrain revealed an AIE‐induced reduction of 24% (±8%) on P56 (24 h post‐AIE), 31% (±4%) on P80 (25 days post‐AIE), 28% (±5%) on P95 (40 days post‐AIE), and 19% (±2%) on P220 (165 days post‐AIE), relative to CONs. Data are presented as % change relative to age‐matched CONs (n = 8/group). *P < 0.05, **P < 0.01, relative to CONs

**Figure 3:**
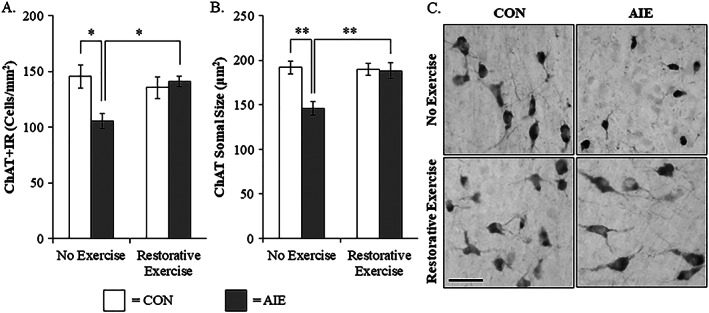
Wheel running restores the adolescent intermittent ethanol (AIE)‐induced loss and somal shrinkage of choline acetyltransferase immunoreactive (ChAT+IR) cholinergic neurons in the adult basal forebrain. A, Modified unbiased stereological assessment revealed a 28% (±5%) reduction of ChAT+IR neurons in the adult (P95) basal forebrain of AIE‐treated subjects, relative to CONs. Running wheel exposure from P56 to P95 did not affect ChAT expression in CONs, but did restore the AIE‐induced loss of ChAT+IR neurons, relative to no exercise AIE subjects. B, Analysis of ChAT+IR neuron somal size revealed a 24% (±4%) reduction in the adult basal forebrain of AIE‐treated subjects, relative to CONs. Wheel running did not affect ChAT neuron somal size in CONs, but did restore the AIE‐induced ChAT+IR neuron somal shrinkage in the adult basal forebrain, relative to the no exercise AIE subjects. C, Representative photomicrographs of ChAT+IR neurons in the adult basal forebrain from CON‐ and AIE‐treated subjects across exercise conditions. Scale bar = 50 μm. Data are presented as mean ± SEM (n = 7‐8/group). *P < 0.05, **P < 0.01

**Figure 4:**
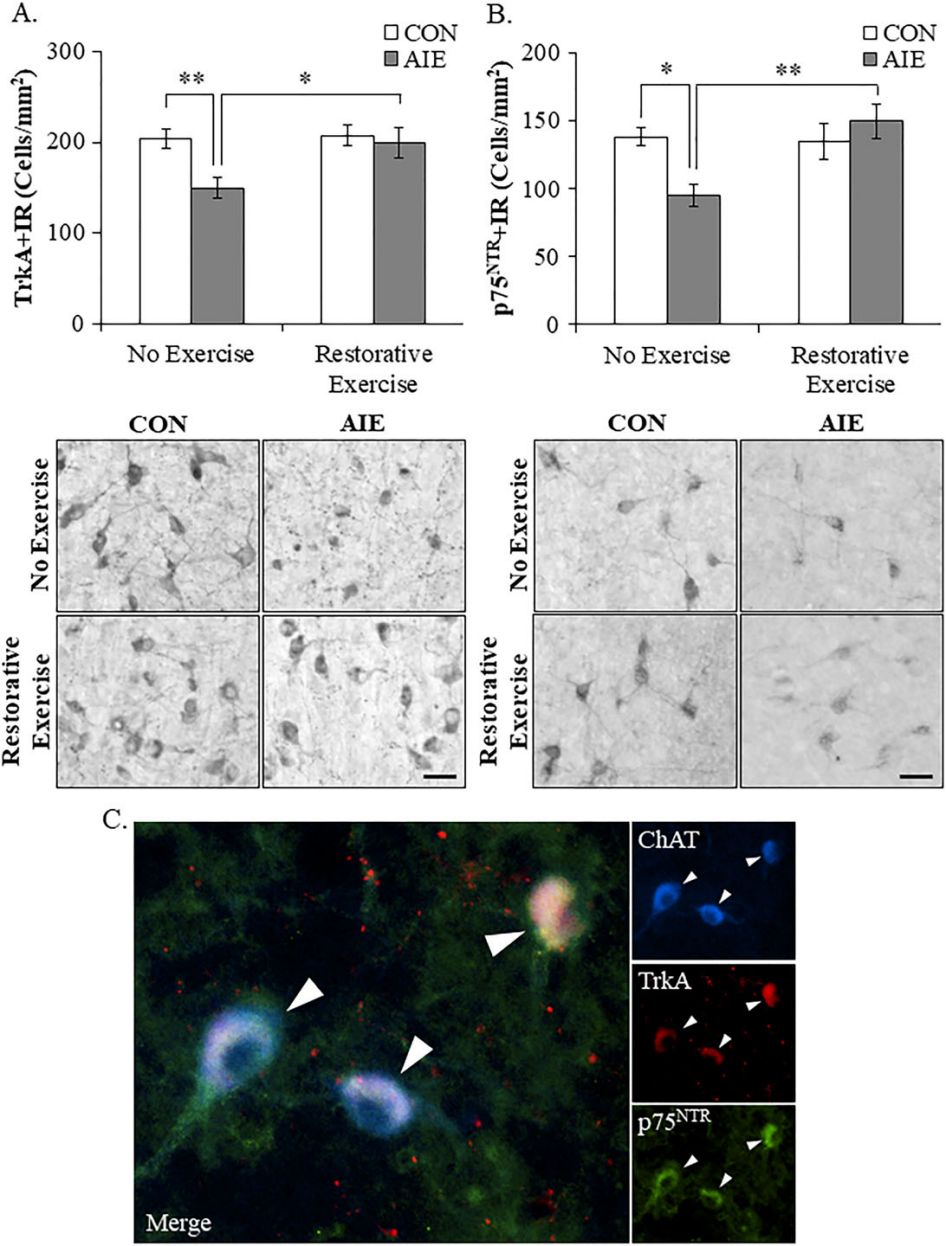
Voluntary exercise exposure following adolescent intermittent ethanol (AIE) restores the loss of tropomyosin receptor kinase A (TrkA)‐ and p75^NTR^‐immunoreactive cells in the adult basal forebrain. A, Modified unbiased stereological quantification of the high‐affinity nerve growth factor (NGF) receptor TrkA in the adult (P95) basal forebrain revealed a 27% (±6%) reduction in AIE‐treated subjects, relative to CONs. Running wheel exposure from P56 to P95 did not affect TrkA expression in CONs, but did restore the AIE‐induced loss of TrkA+IR neurons, relative to no exercise AIE subjects. Representative photomicrographs of TrkA+IR neurons in the adult basal forebrain from CON‐ and AIE‐treated subjects across exercise conditions. Scale bar = 50 μm. B, Modified unbiased stereological quantification of the low‐affinity NGF receptor p75^NTR^ in the adult (P95) basal forebrain revealed a significant 31% (±6%) reduction in AIE‐treated animals, relative to CONs. Wheel running alone did not affect p75^NTR^ expression in CONs, but did restore the AIE‐induced loss of p75^NTR^+IR neurons, relative to no exercise AIE subjects. Representative photomicrographs of p75^NTR^+IR neurons in the adult basal forebrain from CON‐ and AIE‐treated subjects across exercise conditions. Scale bar = 50 μm. C, Immunofluorescent colabeling revealed a high degree of TrkA (red) and p75^NTR^ (green) colocalization with ChAT+IR neurons (blue) in the adult (P95) basal forebrain. Data are presented as mean ± SEM (n = 8/group). *P < 0.05, **P < 0.01

Adolescent intermittent ethanol treatment has previously been shown to reduce ChAT+IR neurons in the basal forebrain^9^, ^10^ that we replicated, and extended finding ChAT+IR neuron loss occurs just after the completion of AIE (ie, P56) that persists into adulthood (ie, P220) (see Figure 2). In a previous study, we reported that exercise initiated at the onset of AIE and continuing throughout experimentation (ie, P24‐P80) prevented the AIE‐induced loss of cholinergic neuron markers in the adult (ie, P80) basal forebrain (see Vetreno and Crews^10^). In the present study using a separate cohort of subjects, we sought to determine if wheel running exposure initiated 24 hours after the conclusion of AIE (ie, P56‐P95) would restore the loss of cholinergic neuron markers in the adult (ie, P95) basal forebrain. We observed a 28% (±5%) reduction of ChAT+IR neurons in the basal forebrain of adult (ie, P95) AIE‐treated subjects (Tukey's HSD: *P* < 0.05), relative to CONs. This reduction of ChAT+IR neurons is similar to the 24% reduction of ChAT+IR cells observed in the adult (ie, P80) basal forebrain from our prior exercise prevention study (see^10^) thereby establishing replicability across experiments. Wheel running initiated 24 hours following the conclusion of AIE from P56 to P95 did not affect ChAT+IR in CONs, but did restore the AIE‐induced loss of ChAT+IR in the adult basal forebrain (Tukey's HSD: *P* < 0.05; see Figure 3A). Assessment of ChAT+IR neuron somal size revealed a significant 24% (±4%) reduction in adult AIE‐treated subjects (Tukey's HSD: *P* < 0.01), relative to CONs. Exercise did not affect ChAT+ somal size in CONs, but recovered the AIE‐induced ChAT+IR neuron somal shrinkage (Tukey's HSD: *P* < 0.01; see Figure 3B/C). Consistent with previous studies,^10^ ChAT+IR neurons colocalized with the trophic factor receptors TrkA and p75^NTR^ (see Figure 4C). Immunohistological assessment of TrkA+IR and p75^NTR^+IR revealed darkly stained cell bodies similar to ChAT+IR. In the present study, we observed a 27% (±6%) reduction of TrkA+IR (one‐way ANOVA: *F*_[1, 14]_ = 12.2, *P* < 0.01; see Figure 4A) and a 31% (±6%) reduction of p75^NTR^+IR (Tukey's HSD: *P* < 0.05; see Figure 4B) in the basal forebrain of adult (ie, P95) AIE‐treated subjects, relative to CONs. Previously, we reported that wheel running from the onset of AIE to the conclusion of experimentation (ie, P24‐P80) prevented the AIE‐induced loss of TrkA‐ and p75^NTR^‐immunoreactive cells in the adult (ie, P80) basal forebrain further establishing reproducibility across experiments (see Vetreno and Crews^10^). In the present study, wheel running from P56 to P95 did not affect TrkA+IR or p75^NTR^+IR cells in CONs, but did restore the AIE‐induced loss of cells expressing TrkA and p75^NTR^ in the adult basal forebrain (see Figure 4). Thus, AIE reduces adult ChAT+IR, TrkA+IR, and p75^NTR^+IR cholinergic neuron numbers that persist from adolescence into adulthood that is restored to constitutive levels by wheel running.

**Figure 5:**
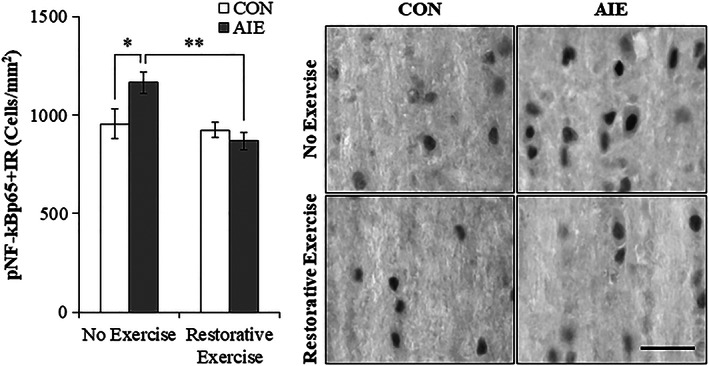
Exercise exposure following adolescent intermittent ethanol (AIE) treatment reverses the increased expression of phosphorylated nuclear factor kappa‐light‐chain‐enhancer of activated B cells p65 (pNF‐κB p65) in the adult basal forebrain. Modified unbiased stereological quantification of pNF‐κB p65+IR cells revealed a 22% (±6%) increase in the adult (P95) basal forebrain of AIE‐treated animals, relative to CONs. Wheel running alone did not affect pNF‐κB p65+IR in CONs, but did block the AIE‐induced increase of pNF‐κB p65+IR cells, relative to no exercise AIE subjects. Scale bar = 50 μm. Data are presented as mean ± SEM (n = 7‐8/group). *P < 0.05, **P < 0.01

Accumulating evidence reveals that AIE treatment causes a persistent upregulation of neuroimmune signaling molecules resulting in adult neuropathology, including loss of ChAT+IR cells.^9^, ^10^, ^31^, ^32^ NF‐κB is a transcription factor known to induce multiple neuroimmune genes,^33^ and phosphorylated NF‐κB p65 has been used to follow neuroimmune activation in brain.^10^, ^15^, ^34^ In a previous study, we reported that exercise initiated at the onset of AIE and continuing throughout experimentation (ie, P24‐P80) prevented the AIE‐induced increase of pNF‐κB p65 in the adult (ie, P80) basal forebrain (see Vetreno and Crews^10^). In the present study, we observed a significant 22% (±6%) increase of pNF‐κB p65+IR in the basal forebrain of adult (ie, P95) AIE‐treated subjects, relative to CONs (Tukey's HSD: *P* ≤ 0.05; see Figure 5). This increase of pNF‐κB p65+IR is similar to the 48% increase of pNF‐κB p65+IR observed in the adult (ie, P80) basal forebrain from our prior exercise prevention study (see Vetreno and Crews^10^) thereby establishing replicability across experiments. In the current study, wheel running from P56 to P95 did not affect pNF‐κB p65+IR in CONs, but resolved the AIE‐induced increase of pNF‐κB p65+IR cells in the adult (ie, P95) basal forebrain (Tukey's HSD: *P* < 0.01). Thus, voluntary exercise initiated following the conclusion of AIE blocked the persistent increase in expression of the neuroimmune marker pNF‐κB p65 and restored the AIE‐induced loss of ChAT, TrkA, and p75^NTR^ in the adult basal forebrain.

### 3.2 Wheel running after AIE does not induce generation of new basal forebrain cholinergic neurons

**Figure 6:**
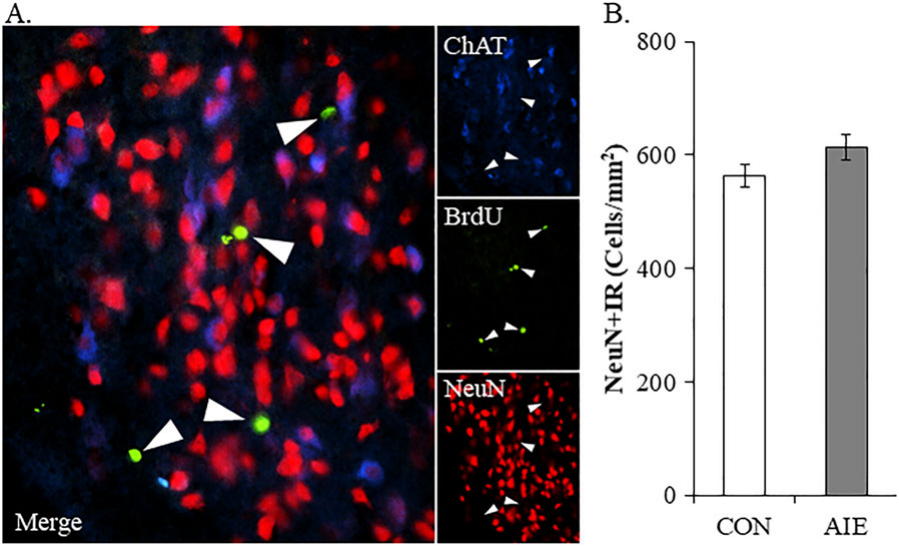
Wheel running after adolescent intermittent ethanol does not induce generation of new basal forebrain cholinergic neurons. A, Immunofluorescent assessment revealed that BrdU (green) did not colocalize with either choline acetyltransferase (ChAT; blue)‐ or NeuN (red)‐immunopositive neurons in the adult basal forebrain. Further, BrdU+IR in the basal forebrain was unaffected by AIE treatment or wheel running exposure. B, Modified unbiased stereological assessment of the neuron marker NeuN in the adult basal forebrain revealed that AIE treatment did not affect NeuN+IR neuron counts, relative to CONs. Data are presented as mean ± SEM (n = 6/group)

Restoration of the AIE‐induced loss of ChAT+, TrkA+, and p75^NTR^+ cells by exercise might represent the generation of new cholinergic neurons from dividing neuroprogenitors. To determine if new cells were formed, we administered BrdU, which is incorporated into DNA during the DNA synthesis phase of cell proliferation, following AIE during exercise exposure. Evaluation of BrdU+IR cells revealed a small population of heterogeneously distributed darkly stained cell nuclei in CON‐ and AIE‐treated basal forebrain. We found that BrdU+IR cell populations were not affected by AIE treatment and that wheel running did not increase BrdU expression in the basal forebrain of AIE‐treated adult subjects (data not shown). In contrast, BrdU+IR cells were significantly decreased in the hippocampal dentate gyrus of AIE‐treated animals (one‐way ANOVA: *F*_[1, 14]_ = 8.5, *P* < 0.05) relative to CON, which was restored to CON levels following exercise exposure (data not shown). We next investigated colocalization of ChAT with BrdU and the neuronal marker NeuN in the adult basal forebrain (see Figure 6A). We did not observe colocalization of BrdU with either ChAT or NeuN in the adult basal forebrain following running wheel exposure. In addition, AIE treatment did not affect NeuN+IR neuron counts suggesting that populations of neurons are not reduced in the adult basal forebrain (see Figure 6B). Together, these data suggest that AIE and exercise do not alter the number of basal forebrain neurons, but do change the number of ChAT+, TrkA+, and p75NTR+ basal forebrain neurons consistent with a change in neuronal phenotype (ie, loss of the cholinergic neuron phenotype).

### 3.3 Adolescent intermittent ethanol‐induced epigenetic modification of the Chat and Trka gene promoters in the adult basal forebrain

**Figure 7:**
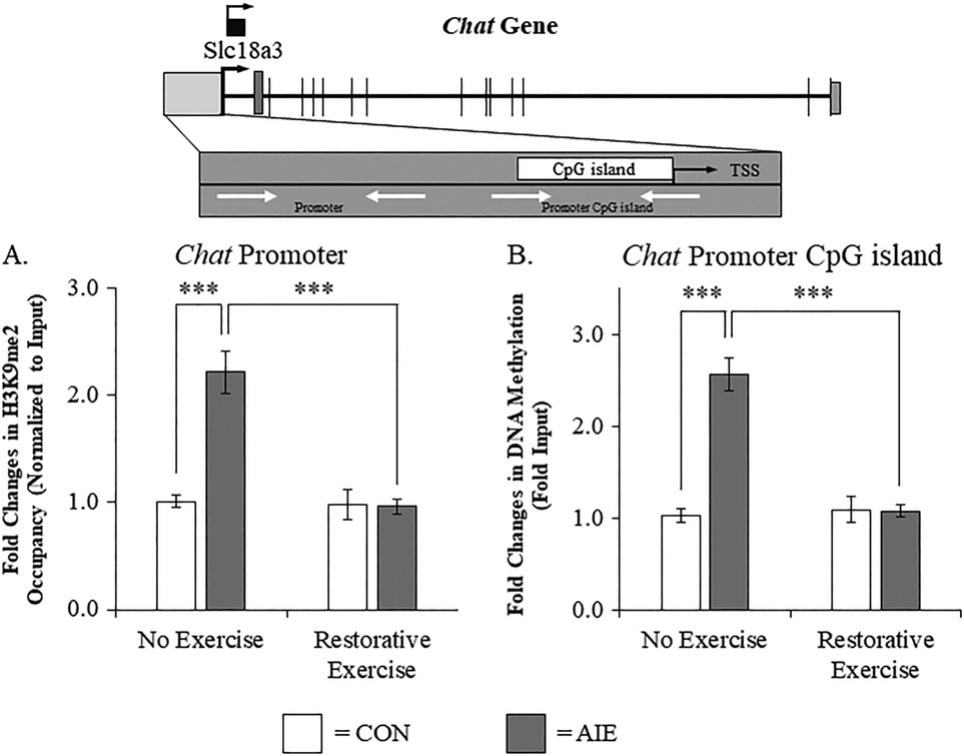
Wheel running recovers the adolescent intermittent ethanol (AIE)‐induced choline acetyltransferase (*Chat*) gene histone and DNA methylation in the adult basal forebrain. A, Chromatin immunoprecipitation assessment revealed that occupancy of histone 3 lysine 9 dimethylation (H3K9me2) at the promoter of the *Chat* gene was increased by approximately 2.2‐fold in the basal forebrain of adult (P95) AIE‐treated animals, relative to CONs. Running wheel exposure from P56 to P95 did not affect levels of H3K9me2 in CONs, but did resolve the AIE‐induced increase of H3K9me2 at the promoter of the *Chat* gene, relative to no exercise AIE subjects. B, Methylated DNA immunoprecipitation (5‐methyl cytosine) assessment revealed that DNA methylation at the CpG island in the *Chat* promoter was increased by 2.5‐fold in the adult (P95) basal forebrain of AIE‐treated animals, relative to CONs. Running wheel exposure from P56 to P95 did not affect *Chat* DNA methylation in CONs, but did resolve the AIE‐induced increase of DNA methylation at the CpG island in the *Chat* promoter, relative to no exercise AIE subjects. Data are presented as mean ± SEM (n = 8‐10/group). ***P < 0.001. TSS, transcription start site

To determine if *Chat* and *Trka* gene expression were altered by an epigenetic mechanism, we assessed histone acetylation and histone methylation within these genes. We found that levels of H3K9me2 at the *Chat* promoter were increased by approximately 2.2‐fold in the adult basal forebrain of AIE‐treated animals, relative to CONs (Tukey's HSD: *P* < 0.01). While wheel running alone did not affect levels of H3K9me2 in CONs, it did prevent the AIE‐induced increase of H3K9me2 at the *Chat* promoter (Tukey's HSD: *P* < 0.01; see Figure 7A). Further, we found that levels of DNA methylation at the CpG island located within *Chat* was increased by approximately 2.5‐fold in the adult basal forebrain of AIE‐treated animals, relative to CONs (Tukey's HSD: *P* < 0.01). Wheel running alone did not affect *Chat* DNA methylation in the CONs, but did prevent the AIE‐induced increase of DNA methylation at the *Chat* promoter CpG island (Tukey's HSD: *P* < 0.01; see Figure 7B). We did not observe an effect of AIE or wheel running on H3K9 acetylation associated with the *Chat* gene (see Supporting Information Figure 1). Thus, AIE treatment causes long‐lasting increases of H3K9me2 associated with the promoter region of the *Chat* gene and increased DNA methylation at the CpG island of the *Chat* promoter, which was restored to CON levels by wheel running. Interestingly, histone acetylation at several locations of the *Chat* gene promoter was not altered by AIE or exercise in the basal forebrain. We also observed that AIE decreased DNA methylation of the gene body of *Chat* that was not affected by exercise (see Supporting Information Figure 1), suggesting that promoter DNA methylation of the *Chat* gene may regulate gene expression.

**Figure 8:**
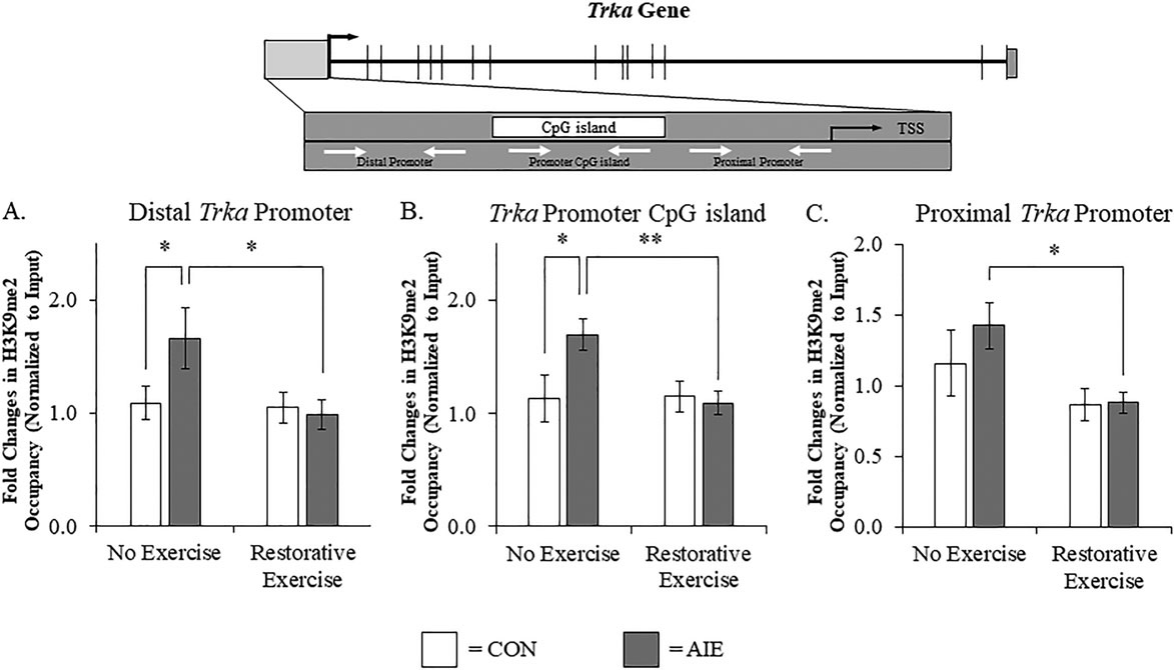
Voluntary exercise exposure recovers the adolescent intermittent ethanol (AIE)‐induced tropomyosin receptor kinase A (*Trka*) gene histone methylation in the adult basal forebrain. A, Chromatin immunoprecipitation (ChIP) assessment revealed that occupancy of histone 3 lysine 9 dimethylation (H3K9me2) of the *Trka* gene at the distal promoter region was increased by approximately 1.7‐fold in the basal forebrain of adult (P95) AIE‐treated animals, relative to CONs. Running wheel exposure from P56 to P95 did not affect levels of H3K9me2 in CONs, but did resolve the AIE‐induced increase of H3K9me2 at the distal promoter of the *Trka* gene, relative to no exercise AIE subjects. B, ChIP assessment revealed that levels of H3K9me2 at the CpG island in the *Trka* promoter were increased by approximately 1.7‐fold in the basal forebrain of adult AIE‐treated animals, relative to CONs. Running wheel exposure from P56 to P95 did not affect levels of H3K9me2 in CONs, but did resolve the AIE‐induced increase of H3K9me2 at the CpG island in the *Trka* promoter, relative to no exercise AIE subjects. C, ChIP assessment revealed that wheel running reduced H3K9me2 at the proximal *Trka* promoter region of adult AIE‐treated animals, relative to no exercise AIE subjects. Data are presented as mean ± SEM (n = 8‐10/group). *P < 0.05, **P < 0.01. TSS, transcription start site

We next assessed whether the AIE‐induced reduction of TrkA is modulated by an H3K9me2 mechanism in the adult basal forebrain similar to what was observed with *Chat* gene expression. Levels of H3K9me2 associated with the distal promoter region of the *Trka* gene (Tukey's HSD: *P* < 0.05; see Figure 8A) and the CpG island in the *Trka* promoter (Tukey's HSD: *P* < 0.05; see Figure 8B) were both increased by approximately 1.7‐fold in the basal forebrain of adult AIE‐treated animals, relative to CONs. While wheel running alone did not affect H3K9me2 at either of these *Trka* promoter regions, it did prevent the AIE‐induced increase of H3K9me2 at the distal *Trka* promoter (Tukey's HSD: *P* < 0.05) and the CpG island in the *Trka* promoter (Tukey's HSD: *P* < 0.01). At the proximal promoter of the *Trka* gene, exercise exposure also decreased levels of H3K9me2 in the AIE‐treated subjects (Tukey's HSD: *P* < 0.05; see Figure 8C). Thus, AIE treatment led to long‐lasting increases of H3K9me2 associated with both *Chat* and *Trka* promoters that were restored by wheel running exposure.

### 3.4 Voluntary exercise restores the reversal learning deficits and increased perseveration on the Morris water maze in AIE‐treated adult rats

**Figure 9:**
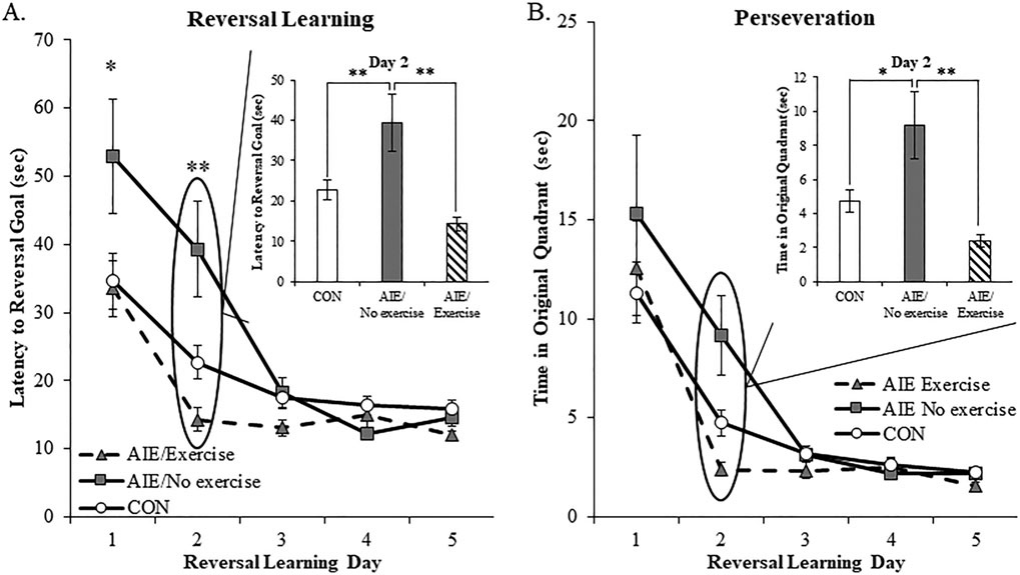
Voluntary exercise exposure following adolescent intermittent ethanol (AIE) restores reversal learning deficits on the Morris water maze. Spatial and reversal learning were assessed in adult subjects using the Morris water maze. Spatial learning was assessed from P82 to P86, and all subjects learned to locate and escape onto the submerged platform to criterion levels by P85. In the CON subjects, performance on the Morris water maze did not differ as a function of exercise exposure, and CON groups were combined to gain statistical power for behavioral assessment. While AIE treatment did not affect the latency to escape or distance traveled during the spatial learning component, all subjects reduced their escape latency across testing days. A, Latency to escape onto the submerged platform during reversal learning (ie, P87 to P91) was increased by 53% during first day and 74% during the second day in the AIE‐treated animals, relative to CONs. Voluntary wheel running blunted the AIE‐induced increase in latency to escape onto the submerged platform during the first and second days of reversal learning, relative to no exercise AIE‐treated animals. B, Assessment of perseveration, defined as time spent in the previous spatial goal quadrant during reversal learning, revealed that AIE treatment increased time spent in the previous spatial goal quadrant by 93% during the second trial of reversal learning, relative to CONs. Wheel running blunted the AIE‐induced increase in perseverative behavior during the second trial of reversal learning, relative to no exercise AIE‐treated animals. Data are presented as mean ± SEM (n = 8‐10/group). *P < 0.05, **P < 0.01

Adolescent intermittent ethanol treatment of mice and rats has previously been found to impair behavioral flexibility inducing reversal, but not spatial learning deficits on the Morris water maze and Barnes maze, respectively.^35^, ^36^ To determine if exercise exposure might recover the reversal learning deficits in adults, AIE‐treated rats were assessed on the Morris water maze. In the CON subjects, performance on the Morris water maze did not differ as a function of exercise exposure (ie, CON‐no exercise vs. CON‐exercise; all *P*s > 0.1), so CON groups were combined to gain statistical power for behavioral assessment. Spatial learning was assessed long after AIE treatment (ie, from P82 to P86), and all subjects learned to locate and escape onto the submerged platform to criterion levels by P85 (CON: 28 ± 4 s; AIE/no exercise: 24 ± 5 s; AIE/exercise: 22 ± 4 s). While AIE treatment did not affect the latency to escape or distance traveled (both *P*s > 0.3) during the spatial learning component, all subjects reduced their escape latency across testing days (main effect of day: *F*_[4, 144]_ = 60.6, *P* < 0.01) indicating that subjects learned the spatial component. Following the completion of spatial learning, reversal learning was assessed 24 hours later beginning on P87. Latency to escape onto the submerged platform during reversal learning was increased by 53% during first day (CON: 35 ± 4 s; AIE: 52 ± 8 s; Tukey's HSD: *P* < 0.05) and 74% during the second day (CON: 23 ± 3 s; AIE: 39 ± 7 s; Tukey's HSD: *P* < 0.01) in the AIE‐treated animals, relative to CONs. Importantly, voluntary wheel running blunted the AIE‐induced increase in latency to escape onto the submerged platform during the first (Tukey's HSD: *P* = 0.08) and second (Tukey's HSD: *P* < 0.01) day of reversal learning (see Figure 9A). Similarly, assessment of perseveration, defined as time spent in the previous spatial goal quadrant during reversal learning, revealed that AIE treatment increased time spent in the previous spatial goal quadrant by 93% during the second trial of reversal learning, relative to CONs (one‐way ANOVA: *F*_[1, 27]_ = 7.2, *P* < 0.05) and that wheel running blunted the AIE‐induced increase in perseverative behavior during the second trial of reversal learning (one‐way ANOVA: *F*_[1, 17]_ = 11.7, *P* < 0.01; see Figure 9B). These studies reveal that AIE‐induced cognitive deficits in reversal learning on the Morris water maze are restored by wheel running similar to restoration of the decreases in ChAT+, TrkA+, and p75^NTR^+ cells in the adult basal forebrain.

## 4 DISCUSSION

**Figure 10:**
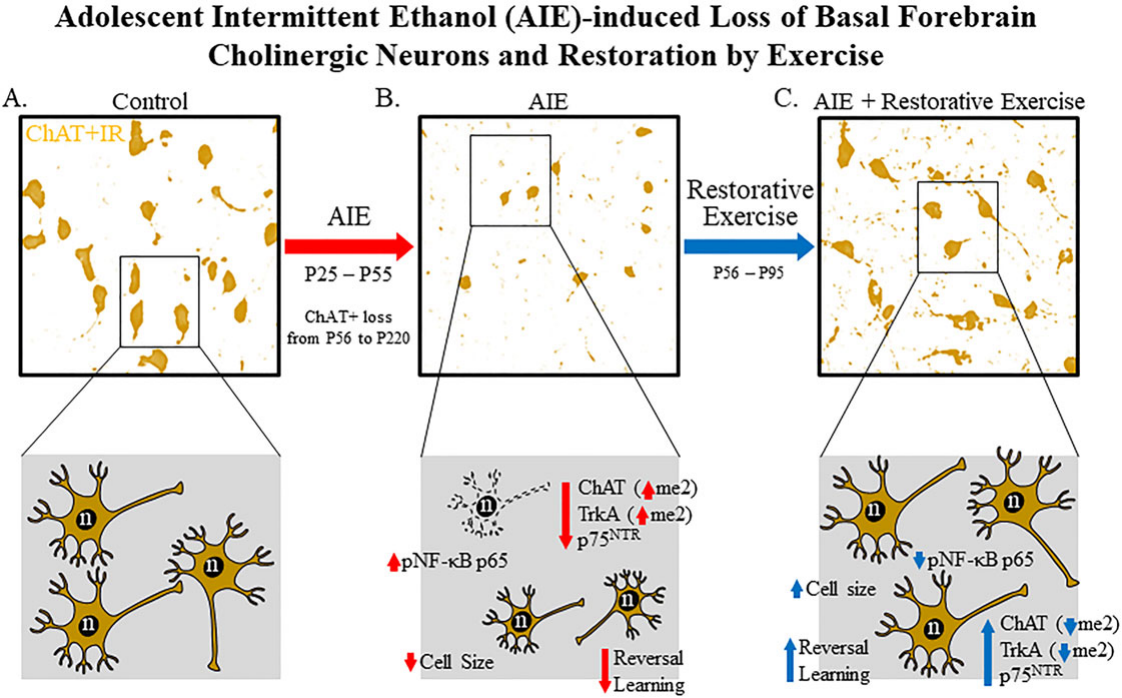
Simplified schematic depicting the proposed mechanism underlying the persistent adolescent intermittent ethanol (AIE)‐induced loss of basal forebrain cholinergic neurons. A, In naïve basal forebrain, cholinergic neurons express choline acetyltransferase (ChAT), the high‐affinity nerve growth factor (NGF) receptor tropomyosin receptor kinase A (TrkA), and the low affinity NGF receptor p75^NTR^. (Top) ChAT+IR neurons in the adult (P95) basal forebrain. (Bottom) Schematic depicting ChAT+IR basal forebrain cholinergic neurons in orange. Note that “n” in the nucleus represents NeuN immunoreactivity. B, AIE causes loss of ChAT+IR neurons in the adolescent (P56) basal forebrain that persists into adulthood (P220). (Top) AIE‐induced loss of ChAT+IR neurons and shrinkage of remaining cholinergic neurons in the adult (P95) basal forebrain. (Bottom) AIE‐induced loss of ChAT‐, TrkA‐, and p75^NTR^‐immunopositive basal forebrain neurons (dashed neuron lacking ChAT [orange]) as well as shrinkage of remaining cholinergic neurons. Note that n in the nucleus represents NeuN+IR, which was unchanged by AIE consistent with loss of the cholinergic phenotype and not cell death. AIE increased dimethylation of lysine 9 of histone 3 (H3K9me2) associated with promoter regions on the *Chat* and *Trka* gene in the adult basal forebrain. AIE increased phosphorylation of the proinflammatory transcription factor NF‐κB p65 in the adult basal forebrain, and neuroimmune signaling might alter gene expression in part through epigenetic mechanisms.^32^ Loss of basal forebrain cholinergic neurons might contribute to the neurocognitive deficits observed in adult AIE‐treated subjects and AIE treatment caused long‐term impairments in reversal learning on the Morris water maze. C, Voluntary wheel running from P56 to P95 restored the AIE‐induced loss of basal forebrain cholinergic neurons. (Top) Restoration of ChAT+IR neurons and shrinkage of remaining cholinergic neurons in the adult (P95) basal forebrain of AIE‐treated subjects. (Bottom) Restoration of ChAT‐, TrkA‐, and p75^NTR^‐immunopositive neurons and reversal of cholinergic neuron shrinkage in adult (P95) basal forebrain of AIE‐treated subjects. Importantly, the restorative effects of exercise do not appear to be because of the generation of new cholinergic neurons as ChAT did not colocalize with BrdU in the adult basal forebrain consistent an AIE‐induced loss of the basal forebrain cholinergic neuron phenotype. Restorative exercise restored to CON levels the AIE‐induced increase of H3K9me2 in promoter regions of both the *Chat* and *Trka* genes as well as the increased expression of pNF‐κB p65 in the adult basal forebrain. This suggests that restorative exercise is a potential treatment modality for maladaptive changes in neural architecture in the basal forebrain that contributes to deficits in learning and cognitive function associated with adolescent binge drinking

In the present study, we discovered that the AIE‐induced reduction of basal forebrain cholinergic neurons is because of the loss of the cholinergic phenotype and not cell death that may involve epigenetic mechanisms. Wheel running exposure (ie, P56‐P95) initiated 24 hours following the conclusion of AIE restored the loss of cholinergic neuron markers (ie, ChAT, TrkA, and p75^NTR^) in the adult basal forebrain. This appears to be mediated by histone (H3K9) and DNA methylation mechanisms. In addition, restorative exercise exposure blocked the AIE‐induced increase of the neuroimmune marker pNF‐κB p65 in the adult basal forebrain. Previous studies find that AIE increases HMGB1, MCP‐1, and other neuroimmune genes that persist into adulthood in multiple brain regions, including the forebrain, cortex, and hippocampus.^15^, ^29^, ^30^, ^36^ Treatment with the inflammagen LPS increases pNF‐κB p65+IR and neuroimmune genes mimicking the AIE‐induced loss of basal forebrain cholinergic neurons and hippocampal neurogenesis,^10^, ^15^ and reduction of NGF, BDNF, and other trophic factors.^10^, ^16^, ^37^ Restoration of ChAT+IR is not because of the generation of new cholinergic neurons since BrdU+IR was rarely observed in the basal forebrain and did not colocalize with ChAT+ neurons. Although it is possible that nondividing or other undifferentiated neuroprogenitors form new cholinergic neurons, the colocalization data coupled with the similar levels of decline for ChAT, TrkA, and p75^NTR^ suggest that all are within the same neurons. Further, we found no loss of NeuN+IR neurons in the adult basal forebrain. Thus, the exercise‐induced restoration of cholinergic neurons in the absence of cholinergic neuron generation suggests that AIE does not cause cell death, but rather the loss of cholinergic phenotype. In previous studies, we found long‐lasting cognitive deficits in decisionmaking, particularly reversal learning deficits.^38^ We extend these studies here to voluntary wheel running restoration of long‐term reversal learning deficits in adult AIE‐treated subjects assessed on the Morris water maze. Together, these data suggest that AIE induces a novel neuroplastic process involving neuroimmune signaling and epigenetic gene silencing that results in the loss of the cholinergic neuron phenotype and cognitive deficits that can be restored by exercise exposure (see Figure 10).

Since AIE decreases basal forebrain ChAT+IR neurons from late adolescence (P56) to adulthood, the observed recovery of cholinergic neurons suggests that AIE does not cause cholinergic neuron death, but rather a loss of the cholinergic phenotype. Survival of cholinergic neurons is dependent upon trophic factors, particularly NGF and BDNF.^39^ Fimbria‐fornix lesion‐induced basal forebrain cholinergic neuron shrinkage and loss of cholinergic neuron markers (ie., ChAT and p75^NTR^) can be recovered by intraventricular infusions of NGF.^12^, ^13^ While NGF is critical for the maintenance and survival of basal forebrain ChAT+ neurons, BDNF is also required for cholinergic neuron survival as decreased number and size of forebrain ChAT+IR neurons is observed in BDNF KO mice.^40^ In some studies, exercise has been reported to induce trophic factors. We found that wheel running initiated during AIE treatment prevented the decrease of hippocampal NGF and concomitant loss of basal forebrain cholinergic neuron markers.^10^ Hall and Savage^41^ reported that wheel running restored loss of forebrain ChAT+IR neurons and blunted the reduction of hippocampal NGF and BDNF in a rodent model of thiamine deficiency. Studies suggest that the AIE‐induced loss of trophic factor receptors, such as the high affinity NGF receptor TrkA, might contribute to the loss of ChAT+IR neurons. Application of the Trk receptor inhibitor K252A blocked the NGF‐induced increase of ChAT protein expression in the N2a cell line^42^ whereas mice lacking TrkA have a reduction in the number and size of ChAT+ neurons.^43^ In the present study, exercise exposure initiated 24 hours following the conclusion of AIE restored the loss of ChAT‐, TrkA‐, and p75^NTR^‐immunopositive cholinergic neuron markers as well as somal shrinkage in the adult basal forebrain, but there was no loss of NeuN+ neurons. This is consistent with the AIE‐induced increased methylation of TrkA suppressing expression and trophic support needed to maintain the cholinergic neuron phenotype. Regardless of the exact mechanism, the exercise‐induced restoration of the AIE‐induce loss of ChAT+IR neurons and cognitive dysfunction suggest that the deficits are not permanent.

Emerging studies suggest that epigenetic modifications contribute to long‐lasting gene expression changes in brain induced by AIE.^44^ We report here that AIE treatment produced long‐lasting increases of H3K9me2 at promoter regions of the *Chat* and *Trka* genes as well as DNA methylation on the CpG island located in the promoter of the *Chat* gene in the adult basal forebrain. Increased H3K9me2 and DNA methylation are processes generally associated with stable transcriptional gene silencing^19^, ^45^ that may contribute to the persistent, long‐term AIE‐induced loss of adult cholinergic neuron markers. Long‐lasting AIE‐induced epigenetic modifications have also been observed in the amygdala and hippocampus of adult rats.^16^, ^21^ Sakharkar et al^16^ reported AIE‐induced reductions of hippocampal neurogenesis and BDNF protein expression that was associated with decreased H3K9/K14 acetylation on the *Bdnf* promoter at exon IV. Interestingly, treatment with the HDAC inhibitor TSA, which increases transcriptional activity through chromatin relaxation, in adulthood restored the reduction of BDNF exon IV acetylation and loss of hippocampal neurogenesis. Work from our laboratory linked neuroimmune activation to AIE‐induced reductions of neurogenesis as LPS treatment mimics while blockade of neuroimmune activation with exercise or the anti‐inflammatory drug indomethacin prevents the AIE‐induced loss of hippocampal neurogenesis.^15^ Together, these two studies fit with neuroimmune‐trophic gene silencing contributing to persistent hippocampal pathology that can be reversed. Epigenetic processes have also been implicated in the regulation of cholinergic genes. In unmanipulated NG108‐15 neuron‐like cell culture, treatment with TSA increased ChAT protein expression.^46^ We report here that exercise exposure restored the aberrant AIE‐induced increase of chromatin and gene methylation to baseline CON levels that paralleled the restoration of cholinergic neuron markers and restoration of neuroimmune activation. Similar to our findings, George et al^47^ reported that wheel running blocks chronic ethanol‐induced epigenetic alterations, cerebrovascular dysfunction, and cognitive impairments in adult mice. Thus, AIE causes long‐term histone methylation of cholinergic genes (ie, *Chat* and *Trka*) in the adult basal forebrain that is restored to baseline levels by exercise exposure.

Previous studies of AIE reveal persistent changes in brain neuroimmune gene expression as well as epigenetic regulation of genes through acetylation and methylation mechanisms that persist long into adulthood and mimic changes observed in the postmortem human alcoholic brain.^14^, ^38^ Recent work suggests that neuroimmune system activation may contribute to epigenetic modifications in response to ethanol. Transgenic mice lacking TLR4 do not show AIE‐induced mPFC changes in histone acetylation and methylation^32^ suggesting that neuroimmune signaling alters gene expression in part through epigenetic mechanisms. Previous studies have linked ChAT+IR neuron loss to neuroimmune signaling through administration of LPS and blockade of AIE‐induced neuroimmune signaling via administration of the anti‐inflammatory drug indomethacin.^10^ In the present study, exercise restored the AIE‐induced increase of pNF‐κB p65 activation, prevented the increased H3K9me3 associated with *Chat* and *Trka* promoters, and loss of cholinergic neurons in the adult basal forebrain. Taken together, these data suggest that neuroimmune signaling is linked to methylation‐induced suppression of *Trka* and *Chat* gene expression.

Basal forebrain cholinergic neurons innervate the cortex, hippocampus, and other brain regions critical for cognitive function,^4^, ^48^ and the AIE‐induced loss of cholinergic neurons may contribute to the neurocognitive deficits observed in adulthood.^29^, ^49^ In the present study, AIE treatment did not affect spatial learning, but did impair reversal learning and increase perseveration in adult rats consistent with previously published studies.^36^ Importantly, we found that exercise exposure restored the AIE‐induced reversal learning deficit and increased perseveration in adulthood. Consistent with the AIE model, a loss of basal forebrain cholinergic neuron markers is observed in human neurodegenerative disorders and alcoholism,^9^, ^50^ which likely contribute to the cognitive dysfunction that accompany these disorders. While the effects of exercise on human basal forebrain cholinergic neurons are unknown, exercise training has been shown to prevent age‐related cognitive decline in humans^51^ consistent with a beneficial effect of exercise on cholinergic neurons of the basal forebrain.

In summary, wheel running initiated 24 hours after the conclusion of AIE restored the loss of cholinergic neurons and concomitant neuroimmune activation. Further, the restorative effects of exercise on the AIE‐induced loss of cholinergic neurons were not because of the formation of new neurons suggesting the loss of the cholinergic neuron phenotype. Interestingly, the AIE‐induced loss of cholinergic neurons was accompanied by a persistent increase in histone methylation of promoter regions of both the *Chat* and *Trka* genes, which was reversed in exercising AIE‐treated subjects. In addition, wheel running restored the AIE‐induced reversal learning deficits and increased perseveration on the Morris water maze. Together, these data implicate a novel neuroplastic process involving neuroimmune and epigenetic mechanisms in the persistent AIE‐induced phenotypic loss of cholinergic neurons in the adult basal forebrain.

## AUTHOR CONTRIBUTIONS

RPV, FTC, and SCP were responsible for the study concept and design. All authors contributed equally to the data preparation and analysis. All authors were involved in drafting and editing the manuscript and have approved the final version for publication.

## Supporting information


**Fig. S1.**
**Effect of adolescent intermittent ethanol (AIE) exposure on choline acetyltransferase (*Chat*) gene acetylation and methylation in the adult basal forebrain.** Top. At the promoter region of the *Chat* gene, neither AIE treatment nor exercise exposure affected (A) DNA methylation, (B) histone 3 lysine 9 acetylation (H3K9ac), or (C) histone 3 lysines 9 and 14 acetylation (H3K9/14Ac) in the adult (P95) basal forebrain. Middle. At the CpG island in the *Chat* promoter, neither AIE treatment nor exercise exposure affected (D) histone 3 lysine 9 dimethylation (H3K9me2), (E) H3K9ac, or (F) H3K9/14Ac in the adult (P95) basal forebrain. Bottom. (G) Methylated DNA immunoprecipitation revealed that DNA methylation at the CpG island of exon 2 in the *Chat* gene was reduced by AIE exposure, relative to CON subjects (Tukey's HSD: *p* < 0.01) that was not affected by exercise exposure.
