# Supplementary material for: Neuroimmune and epigenetic involvement in adolescent binge ethanol‐induced loss of basal forebrain cholinergic neurons: Restoration with voluntary exercise
Source: Addict Biol. 2019 Feb 18;25(2):e12731. doi: 10.1111/adb.12731 (PMC6698434; doi:10.1111/adb.12731)
Supplement: Supplementary file 1 — Fig. S1. Effect of adolescent intermittent ethanol (AIE) exposure on choline acetyltransferase (Chat) gene acetylation and methylation in the adult basal forebrain. Top. At the promoter region of the Chat gene, neither AIE treatment nor exercise exposure affected (A) DNA methylation, (B) histone 3 lysine 9 acetylation (H3K9ac), or (C) histone 3 lysines 9 and 14 acetylation (H3K9/14Ac) in the adult (P95) basal forebrain. Middle. At the CpG island in the Chat promoter, neither AIE treatment nor exercise exposure affected (D) histone 3 lysine 9 dimethylation (H3K9me2), (E) H3K9ac, or (F) H3K9/14Ac in the adult (P95) basal forebrain. Bottom. (G) Methylated DNA immunoprecipitation revealed that DNA methylation at the CpG island of exon 2 in the Chat gene was reduced by AIE exposure, relative to CON subjects (Tukey's HSD: p < 0.01) that was not affected by exercise exposure. [file ADB-25-e12731-s001.docx]

Supporting Information


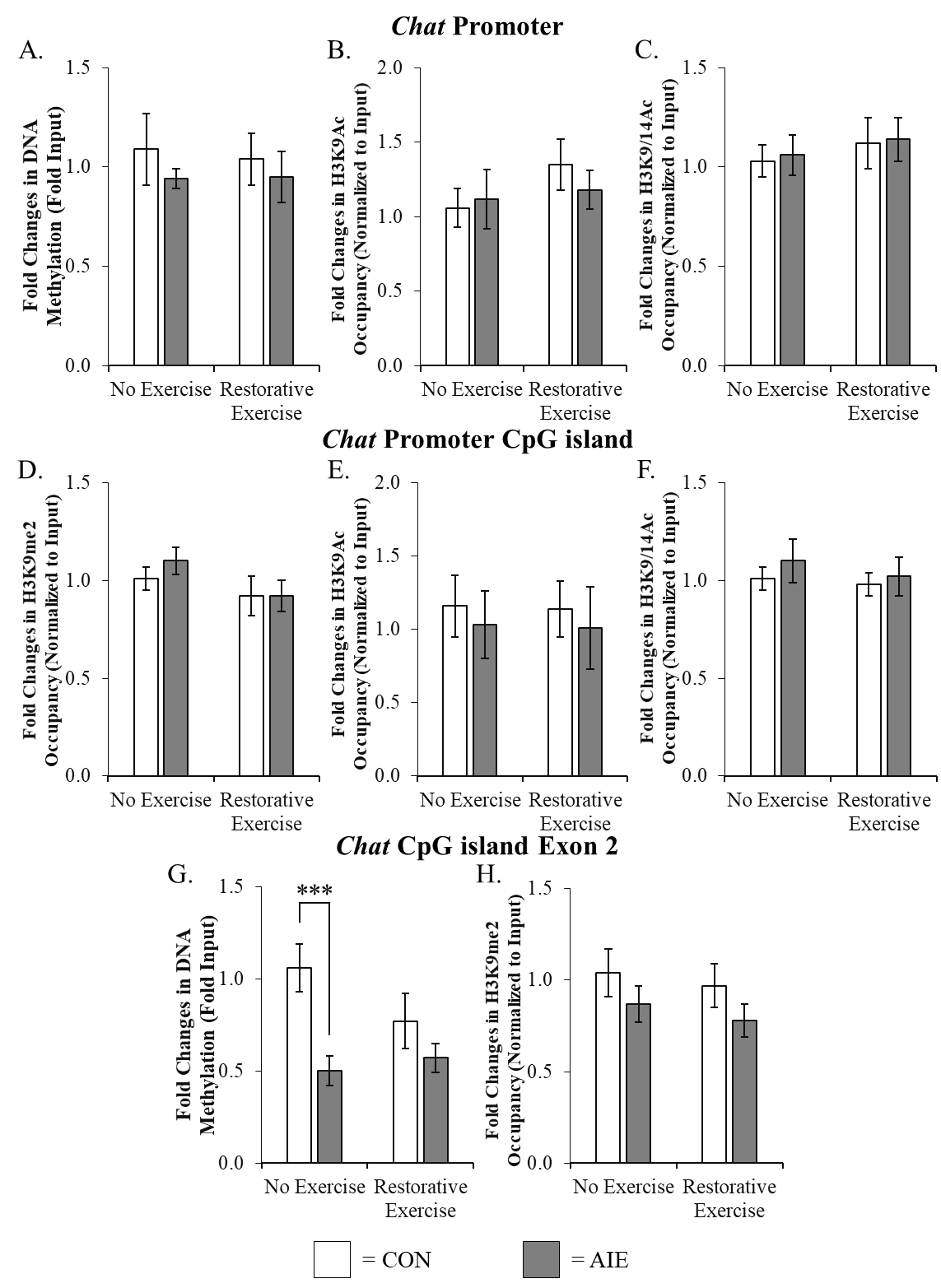


**Fig. 1. Effect of adolescent intermittent ethanol (AIE) exposure on choline acetyltransferase (*Chat*) gene acetylation and methylation in the adult basal forebrain.** Top. At the promoter region of the *Chat* gene, neither AIE treatment nor exercise exposure affected (A) DNA methylation, (B) histone 3 lysine 9 acetylation (H3K9ac), or (C) histone 3 lysines 9 and 14 acetylation (H3K9/14Ac) in the adult (P95) basal forebrain. Middle. At the CpG island in the *Chat* promoter, neither AIE treatment nor exercise exposure affected (D) histone 3 lysine 9 dimethylation (H3K9me2), (E) H3K9ac, or (F) H3K9/14Ac in the adult (P95) basal forebrain. Bottom. (G) Methylated DNA immunoprecipitation revealed that DNA methylation at the CpG island of exon 2 in the *Chat* gene was reduced by AIE exposure, relative to CON subjects (Tukey’s HSD: p < 0.01) that was not affected by exercise exposure. Interestingly, we observed an unexpected trend for wheel running to reduce DNA methylation at the CpG island at Exon 2 of the *Chat* gene. (H) Levels of H3K9me2 at the CpG island of exon 2 in the *Chat* gene were unaffected by either AIE treatment or exercise exposure. Data are presented as mean ± SEM (n = 8-10/group). *** *p* ≤ 0.001.
